# Population specificity in mandibular sexual dimorphism: validation and development of forensic models for application in the contemporary Malaysian population

**DOI:** 10.1007/s00414-026-03924-9

**Published:** 2026-07-27

**Authors:** Choy Ker Woon, Zuzana Obertova, Ambika Flavel, Lauren Swift, Ng Wei Lin, Eric Chung, Daniel Franklin

**Affiliations:** 1 Department of Anatomy, Faculty of Medicine, Universiti Teknologi MARA, Sungai Buloh Campus, Selangor, 47000 Malaysia; 2 Centre for Forensic Anthropology, School of Social Sciences, The University of Western Australia, Crawley Western Australia, 6009 Australia; 3 Department of Biomedical Imaging, Faculty of Medicine, Universiti Malaya, Kuala Lumpur, Malaysia

**Keywords:** Sex estimation, Mandible, Forensic anthropology, Population-specific standards, Malaysia population

## Abstract

**Introduction:**

Sex estimation is a critical part of the forensic anthropological assessment. The mandible, due to its robust nature and sexually dimorphic traits, is often a alternative region for sex estimation, particularly in cases where other skeletal are incomplete or absent. Population-specific studies are necessary to refine and enhance the accuracy of forensic methodologies. This study, therefore, aims to quantify mandibular sexual dimorphism and explore population variances, validate existing discriminant functions, and develop novel models for sex estimation specific to the Malaysian population.

**Methodology:**

The sample comprised cranial multi-detector computed tomographic scans of 213 male and 202 female Malaysian individuals. Following 3D volume rendering, nine mandibular landmarks were acquired using OsiriX, from which a total of 10 linear inter-landmark and one angular measurement were calculated. The data were then analysed using descriptive statistics and discriminant function analyses employing jackknife validations of classification results.

**Results:**

All measurements were sexually dimorphic except for the mandibular angle. Bi-condylar breadth, ramus height, maximum mandibular length, and bi-coronoid breadth were the most dimorphic. Application of foreign discriminant functions to the Malaysian sample resulted in reduced accuracy and a greater sex bias compared to the original studies. The Malaysian specific univariate and multivariate discriminant functions ranged from 62.7 to 83.60% classification accuracy with associated 0.06 to -5.01% sex bias values.

**Conclusion:**

The findings emphasize the importance of validating and developing population-specific mandibular models for accurate sex estimation in the contemporary Malaysian population, while future research should expand multi-ethnic datasets to further enhance forensic identification reliability.

## Introduction

Sex estimation is a cornerstone of forensic anthropology, providing an essential initial part of the biological profile that potentially significantly narrows the available pool of potential identities for adult remains [1]. Accurate estimation of sex also supplements the accuracy of estimations of age, stature and population affinity, thereby strengthening the overall process of human identification and demographic reconstruction in both forensic and bioarchaeological contexts [2]. Among the various skeletal elements, the pelvis are y recognized as the most suitable structures for the assessment of sexual dimorphism, followed by long bones and skull [3]. However, these elements are often fragmented, incomplete or missing, especially in catastrophic events [4]. The human mandible is frequently recovered intact or sufficiently preserved due to its dense cortical bone. Consequently, mandibular sexual dimorphism has been quantified across global populations as alternative skeletal region for sex estimation [5–7].

Sex estimation using the mandible is undertaken using morphoscopic or morphometric approaches. Amongst the most common measurements used to derive statistical models to classify sex are the mandibular angle, bicondylar breadth, bigonial breadth, ramus height and symphysis height [8–10]. Morphoscopic approaches typically involve the quantification of readily identifiable dimorphic traits, including gonial flaring, symphysis height and ramus flexure [11–14]. Irrespective of the type of data analysed, it is now well established that the expression and magnitude of sexual dimorphism is population-specific and thus not universally globally applicable [15]. This highlights the necessity of validating and developing population-specific forensic standards.

In Malaysia, a multi-ethnic nation comprising Malay, Chinese, Indian and Indigenous groups, the forensic anthropologist must work within the constraints of unique challenges in around standardizing biological profiling [16]. Further, in tropical environments such as Malaysia, characterised by high humidity, elevated temperatures, and accelerated decomposition rates, human remains are frequently recovered in a fragmented and disarticulated state rather than as complete skeletons [17]. These are evidenced in Wang Kelian mass graves, where large-scale forensic recovery revealed incomplete skeletal remains where sometimes, only the mandible is found in isolation [18], highlighting the practical challenges of relying on complete cranial or pelvis structures for sex estimation. In such scenarios, anatomically robust and discrete elements, particularly the mandible, are more likely to persist and be available for forensic analysis. Despite growing forensic casework in Malaysia [19], there remains a paucity of robust statistical predictive models for the mandible optimised for the contemporary Malaysian population. Therefore, it is essential to assess the applicability of the mandible for sex estimation in the Malaysian population, whilst concurrently furthering the understanding of population variation relative to forensic practice.

In modern forensic practice, the role of imaging modalities, particularly computed tomography (CT), is well recognized in post-mortem investigations [20]. The application of 3D virtual models as alternative for physical specimens has become an invaluable tool that enables forensic scientists to access contemporary and high-resolution data [21, 22]. The generally non-significant discrepancy between traditional osteometric and volume-rendered MSCT inter-landmark measurements demonstrates the reliability of MSCT-derived data for applications traditionally based on dry bone analysis [23]. This is particularly crucial in regions such as Malaysia with limited or non-existent local osteological collections available for research.

The aims of the present study are: **(i)** to quantify the expression and magnitude of sexual dimorphism in the mandible in Malaysian cranial CT scans; **(ii)** to explore the accuracy of existing foreign statistical models for estimating sex based on mandibular measurements; and **(iii)** to develop statistical models specific to the contemporary Malaysian population for sex estimation of the mandible.

## Materials and methods

### Materials

The present study analysed measurements acquired in cranial three-dimensional volume rendered cranial MDCT scans of 415 adult individuals (213 males; 202 females). The sample comprised patients presenting for routine cranial clinical evaluation between 2018 and 2024 at the Department of Biomedical Imaging, University of Malaya Medical Centre, Kuala Lumpur, Malaysia. Scans were anonymised, with only sex, age and ethnicity data retained. The mean male age was 40.98 years (SD = 16.14 years) with a range of 18–80 years; mean female age was 48.00 years (SD = 19.9 years) with a range of 18–83 years. The ethnicity was recorded as Malay (*n* = 144), Chinese (*n* = 137) and Indian (*n* = 134). the proportional representation reflects the distribution of major ethnic groups in the contemporary Malaysian population.

Individuals with obvious pathological or traumatic changes to the mandible were excluded if it affected normal morphology and/or accurate location of measurement landmarks. Research ethics approval was granted by the University of Teknologi MARA [Ethic number: REC/10/2022 (PG/EX/52)] and University of Malaya Institution of Ethics Committee (Ethics number: 201944-7288) and in accordance with the Declaration of Helsinki.

### Visualisation and measurement acquisition

**Fig. 1:**
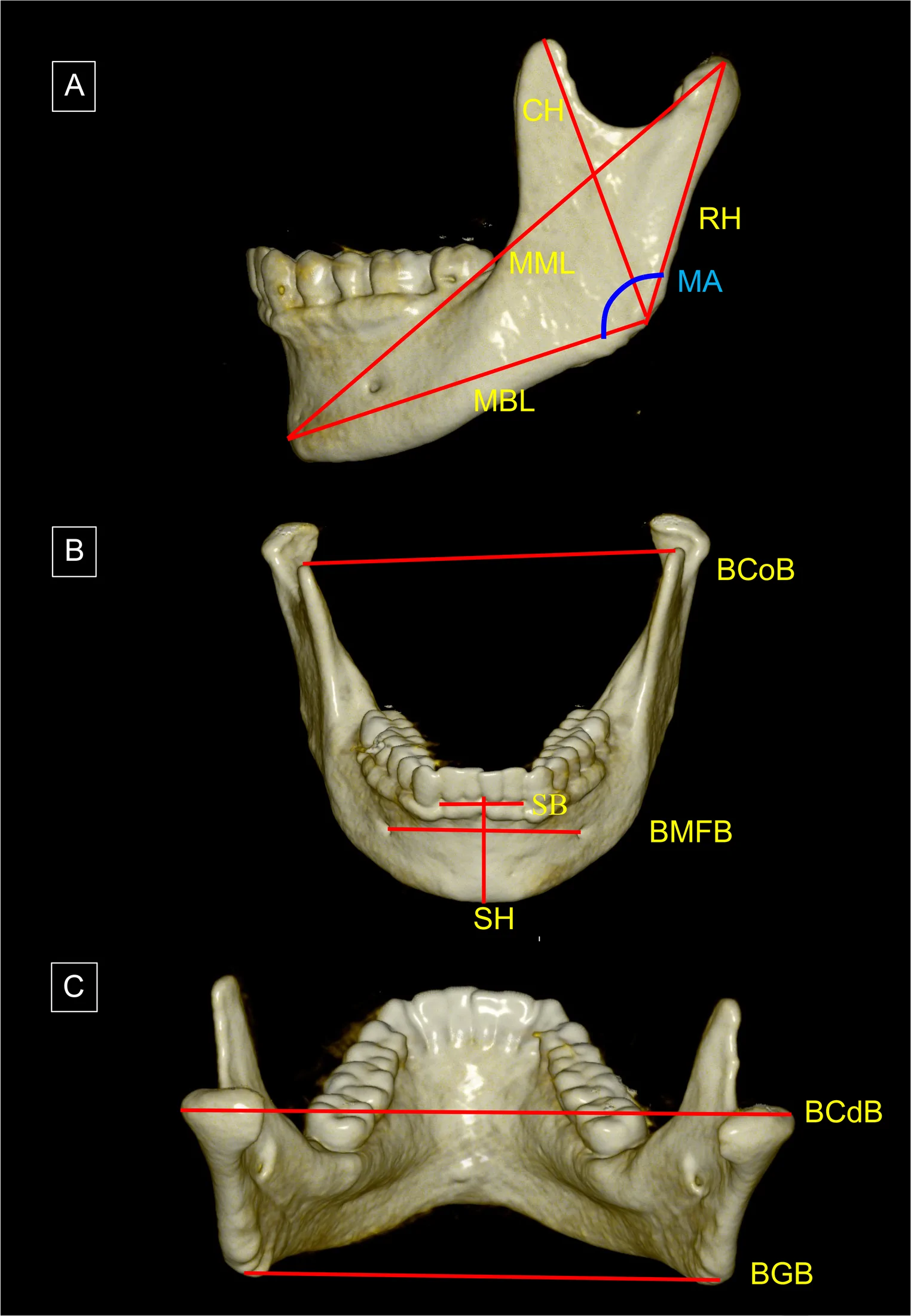
Mandibular measurements taken in this study: (**A**) Lateral, (**B**) Anterior, and (**C**) Posterior view

Cranial imaging was performed on a Phillips Brilliance 64, with an average slice thickness of 0.75 mm. CT images were extracted from the Picture Archiving and Communication System (PACS) in DICOM format. Following 3D volume rendering, the 3D coordinates of nine anatomical landmarks were acquired by CKW using *OsiriX* (version 4.1.1–64 bit) as defined in Table 1. In total, 10 linear and one angle measurement were calculated from the landmarks as defined and illustrated in Table 2; Fig. 1, respectively.

**Table 1:** Definition of landmarks used in the present study; see Fig. 1 for illustrations

No	Landmark	Abbreviation	Definition
1.	Coronion	co	The most superior point on coronoid process
2.	Condylion superior	cs	The most superior point on condyloid process
3.	Condylion laterale	cdl	The most lateral point on the condyloid process
4.	Gonion	go	The most lateral external point at the junction of the horizontal and ascending rami of the lower jaw
5.	Mental foramen	men	Small opening on the anterolateral surface of the mandible below the second premolar tooth.
6.	Lateral infradentale	lid	The midpoint of a line tangent to the anterior margin of the alveolae of lateral incisor and canine teeth
7	Gnathion	gn	The midpoint of the lower border of the mandible in the sagittal plane
8	Pogonion	pg	The most projecting point of the chin in the sagittal line
9	Infradentale	id	The midpoint of a line tangent to the anterior margins of the alveolae of the two mandibular central incisor teeth

**Table 2:** Definitions of the measurements used in the present study; see Table 1 for landmark definitions

No	Measurement	Abbreviation	Landmarks	Definition
1.	Bicondylar breadth	BCdB	cdl–cdl	The transverse distance between the two condylar processes of the mandible in posterior view.
2.	Bicoronoid breadth	BCoB	co–co	The transverse distance between the two coronoid processes of the mandible in anterior view.
3.	Bimental foramina breadth	BMFB	men-men	The distance between the mental foramina in anterior view.
4.	Bigonial breadth	BGB	go–go	The distance between the two gonion points of the mandible in posterior view.
5.	Ramus height	RH	cs–go	The distance from the highest point of the condyloid process to gonion in lateral view.
6.	Coronoid height	CH	co–go	The distance from the highest point of the coronoid process to gonion in lateral view.
7	Mandibular Body length	MBL	go–pg	The horizontal distance from gonion to mental protuberance in lateral view.
8	Symphysis height	SH	gn–id	The vertical distance from the inferior border of the mandible to the most superior point of the mental symphysis in anterior view.
9	Symphysis breadth	SB	lid–lid	The maximum transverse distance at the level of the mental symphysis in anterior view.
10	Maximum mandible length	MML	cs–pg	The total length of the mandible, measured from the condyloid process to the most anterior point of the mental symphysis in lateral view.
11	Mandibular angle	MA	cs-go-gn	The angle formed between the lower border of the mandible and the posterior border of the mandibular ramus in lateral view.

References: (i) Franklin et al. [6]; (ii) Dong et al. [30]; (iii) Bertsatos et al. [31]

### Statistical analyses

A precision test was performed to statistically assess intra-observer error. Six mandibles (three female and three male) were randomly selected from the sample and measured on six separate occasions, with at least one day between repetitions to limit data recall. Intra-observer error is quantified as follows: (i) technical error of measurement (TEM), which is a measure of the standard deviation (SD) between repeat measurements of the same specimen obtained by the same individual; (ii) relative technical error of measurement (rTEM), which is a percentual expression of TEM; and **(iii)** the coefficient of reliability (R), which expresses the level of variance that cannot be attributed to measurement error [24, 25]. For linear measurements, *R* > 0.75 and rTEM < 5% were considered acceptable [26–28].

Prior to performing any analyses, the data were assessed for outliers by identifying measurements that were: (i) unusually large or small according to boxplots; or (ii) had z-scores greater than three SDs. Outliers were rectified if they were found to be due to mislabelling of mandible landmarks or miscalculation, after which they were included in further analyses. Cases representing extreme metric variations, potentially due to morphologically unidentified pathological alterations, were excluded from further analyses as not to skew the normal distribution and thus disproportionally influence the subsequent discriminant function and resulting estimates.

Following assessment of outliers, descriptive statistics were calculated and mean male and female measurement values were compared using analysis of variance (ANOVA) to evaluate the magnitude of sexual dimorphism. To formulate population-specific models for sex estimation, the measurements were then analysed using direct univariate (dDF), multiple variate (mDF), and stepwise (sDA) discriminant function analyses.

Measurement combinations were selected relative to mandibular regions that are known to be sexually dimorphic and have a greater likelihood of postmortem preservation. Direct multivariate discriminant functions were derived based on five distinct possibilities of mandibular preservation and two combinations of the most dimorphic measurements [6]:

- **mDF #1**: Complete mandible.
- **mDF #2**: Half-mandible from the ramus to the chin.
- **mDF #3**: Body only.
- **mDF #4**: Ramus only.
- **mDF #5**: Fragment of the mandibular body.
- **mDF #6**: Three most dimorphic measurements (by F-value).

For the discriminant functions the following data are reported: (i) group centroid; (ii) cross-validated classification accuracy using a jackknife leave-one-out approach; (iii) sex bias values calculated as the difference between the classification accuracy of males relative to females; **(iv)** average posterior probability (PP) values for correctly classified individuals within 0.80 to 1.0 percentile ranges, indicate the model’s confidence in its classifications, with higher probabilities reflecting greater certainty in accurately distinguishing between sexes; and **(v)** area under the curve (AUC) from receiver operator curves (ROC) with 95% confidence interval (CI) to assess sensitivity and specificity of the model performance across different samples.

All statistical analyses were performed using SPSS 19.0 [29].

## Results

### Intra-observer precision

TEM values for all measurements were below 1, ranging from 0.607 mm (Bicoronoid breadth to 0.973 mm (bigonial breadth). The rTEM values for all measurements are below 5%. The measurement with the highest rTEM value is symphysis height (1.86%) and the measurement associated with the lowest rTEM value is maximum mandible length (0.53%). All measurements have associated R values above 0.75, reflecting acceptable reliability, in which the measurements with the lowest R values are ramus height (0.836) and the highest were coronoid height (0.977).

### Descriptive statistics and one-way ANOVA

Descriptive statistics for both sexes, along with results of group comparisons, are summarized in Table 3. Male mean values were consistently higher than those of females. One-way ANOVA indicated significant differences between male and female measurement values, with the exception of the mandibular angle. Bigonial breadth, ramus height and maximum mandibular length were found to be the most dimorphic as evidenced by their respective F-values (Table 3).

**Table 3:** Descriptive statistics including mean, standard deviation and range (in mm). Results of the one-way ANOVA (F and P values) provided in descending order according to F value.

^#^Measurements	Male (*n* = 213)				Female (*n* = 202)				F	*p*-value
	Mean	SD	Min	Max	Mean	SD	Min	Max		
BGB	92.55	6.15	77.83	112.23	81.98	5.54	76.83	106.27	172.76	< 0.0001
RH	63.02	5.10	48.32	72.62	54.20	4.93	37.16	67.48	138.38	< 0.0001
MML	129.06	6.21	98.51	142.33	119.27	5.56	99.40	120.07	137.52	< 0.0001
BCoB	91.90	6.26	78.36	117.77	85.08	5.57	72.47	102.76	128.41	< 0.0001
CH	65.31	6.19	45.03	78.65	56.94	5.44	46.25	72.93	87.72	< 0.0001
MBL	89.25	5.32	73.61	99.78	81.55	5.26	65.54	97.40	83.78	< 0.0001
BCdB	118.52	7.50	107.31	143.23	109.55	6.57	104.49	134.07	72.39	< 0.0001
SH	36.96	4.11	21.09	45.95	31.75	3.84	20.15	39.95	32.23	< 0.0001
SB	26.71	2.64	19.39	32.21	22.43	2.11	18.27	27.60	29.51	< 0.0001
BMFB	48.61	3.01	42.43	59.59	41.23	3.27	35.63	55.11	20.07	< 0.0001
MA	122.70	5.69	98.88	136.14	119.72	5.45	97.88	133.70	0.01	0.94

#Key to measurements in Table 2

### Validation of Existing Discriminant Functions Using Malaysian Mandibular Measurements

The malaysian mandibular measurements were entered into discriminant functions from other populations (Table 4). Applying the ‘Black’ (Bantu-speaking) South African model of Franklin et al. [6] to the Malaysian sample resulted in a decrease in accuracy from 84.0% in the reference to 74.68% in the Malaysian population, with an associated sex bias of 7.91%. When using models developed for a Chinese Han population of Dong et al. [30], a decrease in accuracy from 84.9% in the original sample to 61.84% in the Malaysian sample was observed, with an associated sex bias of -66.44%. Classification models for a Greek population from Bertsatos et al. [31] demonstrated a reported accuracy of 85.0%, which decreased to 72.4% in the Malaysian population, with a sex bias of 13.12% (Table 4) .

**Table 4:** Comparison of cross validation accuracy and sex bias values of other populations when applied to contemporary Malaysian population

Authors & reference	Population	Discriminant functions	Group centroids	Accuracy (sex bias) in original population	Accuracy (sex bias) in Malaysian population
Franklin et al. [6]	South Africa	(RH x 0.095) + (SH x 0.059) + (CHX 0.080) + (BGB x 0.050) + (BCdB x -0.004) + (SB x 0.045) + (CH x 0.037) + (ML x -0.019) + (-25.805)	♀ 0.928, ♂ -1.061	84.00% (-1.43%)	74.68% (7.91%)
Dong et al. [31]	Chinese Han	BCoB x 0.008) + (BGB x 0.049) + (RH x 0.127) + (MML x 0.087) − 24.711	♀− 1.05, ♂0.873,	84.90% (-3.50%)	61.84% (-66.44%)
Bertsatos et al. [32]	Greek	(RH x 0.066) + (CH x 0.095) + (MML x 0.109) − 22.949	♀− 1.05, ♂0.878	77.1% (3.00%)	73.91% (13.12%)

### Discriminant function analyses

#### Direct univariate discriminant function analysis

A series of univariate discriminant function analyses (dDF #1–4) were performed to assess sex classification accuracy of the individual mandibular measurements in the Malaysian population. Only variables with an accuracy of at least 70% and a sex bias of less than 5% are included in Table 5. The most accurate single variable was bigonial breadth (dDF #2) at 75.9% with a sex bias of -2.58%. Additionally, 56.36% of males and 56.40% of females were classified with a posterior probability (PP) greater than 80%. The next most accurate variable was maximum mandibular length (dDF #4), with an accuracy of 74.7% and a sex bias of 3.75%, followed by ramus height (dDF #3) at 71.8% with a sex bias of -3.81%. Bicoronoid breadth (dDF #1) correctly classified 70.4% of individuals with a sex bias of -2.77% (Table 5).

**Table 5:** Performance of the direct, multiple, and stepwise cross-validated discriminant analyses

		Cross validated Accuracy (%)	Correctly Assigned		Sex Bias (%)	Posterior Probabilities 0.80 -1.00 (%)	
	Measurement(s) involved		Male	Female		Male	Female
**Direct Single Variable**							
dDF #1	BCoB	70.40	147/213	145/202	-2.77	40.35	34.65
dDF #2	BGB	75.90	159/213	156/202	-2.58	56.36	56.40
dDF #3	RH	71.80	149/213	149/202	-3.81	42.32	42.33
dDF #4	MML	74.70	163/213	147/202	3.75	49.92	47.30
**Direct Multiple Variables**							
mDF #1: Complete Mandible	BCoB, BCdB, BGD, BMFB, RH, CH, MBL, SH, SB, MML, MA	82.40	175/213	167/202	-0.51	76.02	72.51
mDF #2: Half Mandible	RH, CH, MA, SH, ML	77.60	166/213	156/202	0.71	45.50	40.74
mDF #3: Body only	SH, SB, BMFB	67.20	138/213	141/202	-5.01	21.00	15.67
mDF #4: Ramus only	RH, CH, GA	74.00	154/213	153/202	-3.44	22.32	32.33
mDF #5: Body fragment	SH	62.70	133/213	127/202	-0.43	45.51	51.28
mDF #6: Three most Dimorphic	BGB, RH, MML	80.70	172/213	163/202	0.06	52.47	53.70
**Stepwise Multiple Variables**							
sDF #1	GA, BGB, RH, MBL, MML	83.60	176/213	171/202	-2.02	72.35	68.82

# Key to measurement in Table 2

#### Direct multivariate and stepwise discriminant function analysis

To assess the accuracy of various measurement combinations applicable to mandibles in different preservation states, a series of direct multiple and stepwise variable discriminant analyses were performed (mDF #1–5; sDF #1) as outlined in Table 5. Among direct multivariate discriminant analyses, cross-validated classification accuracy for these models ranged from 62.7% (mDF #5, mandibular fragment) to 82.4% (mDF #1, complete mandible), with corresponding sex bias values between − 5.01% (mDF #3, mandibular body) and 0.71% (mDF #2, half mandible) (Table 5). When using mDF #1 (complete mandible) 76.02% of males and 72.51% of females were classified with a posterior probability (PP) greater than 80%. The model with three most dimorphic measurements (mDF #6) achieved an accuracy of 80.7% and a sex bias of 0.06%, with 52.47% of males and 53.70% of females classified with posterior probability (PP) greater than 80%.

The stepwise multiple variable discriminant model (sDF #1) with mandibular angle, bigonial breadth, ramus height, mandibular body length, and maximum mandibular length resulted in an accuracy of 83.6%, with a sex bias of -2.02%; 72.35% of males and 68.82% of females were classified with posterior probability (PP) greater than 80%.

#### Discriminant equations, group centroid and ROC/AUC analysis

The direct and stepwise discriminant equation derived for the Malaysian population are shown in Table 6. The results of the ROC/AUC analysis aligned with the trend observed in the classification accuracy and posterior probability data in Table 5. Among direct single variable models, the highest AUC was 0.824, corresponding to bigonial breadth (dDF #2). Among direct multivariate and stepwise discriminant functions, the three models that demonstrated the highest AUC values were the same ones that achieved the highest classification accuracy (mDF #1, mDF #6 and sDF #1), with AUC value of 0.900, 0.864 and 0.893, respectively.

**Table 6:** Discriminant equations and group centroids by sex

Functions	Equations: unstandardised coefficients and constants	Group centroid		ROC/AUC Analysis	
		Male	Female	AUC	SE
**Direct Single Variable**					
dDF #1	−16.272 + (0.169×BCoB)	0.395	-0.417	0.750	0.024
dDF #2	−16.334 + (0.170×BGD)	0.628	-0.663	0.824	0.020
dDF #3	−11.397 + (0.199×RH)	0.562	-0.593	0.798	0.022
dDF #4	−20.802 + (0.169×MML)	0.561	-0.591	0.805	0.022
**Direct Multiple Variables**					
mDF #1: Complete Mandible	−35.796 + (0.181×MA) + (0.007×BCoB) + (− 0.033×BCdB) + (0.094×BGD) + (− 0.056×BMFB) + (0.256×RH) + (0.039×CH) + (0.184×MBL) + (0.011×SH) + (0.067×SB) + (− 0.188×MML)	0.91	-0.96	0.900	0.015
mDF #2: Half mandible	−23.634 + (0.135×RH) + (0.035×CH) + (0.061×MA) + (0.008×SH) + (0.051×MML)	0.65	-0.68	0.826	0.020
mDF #3: Body only	−15.231 + (0.164×SH) + (0.252×SB) + (0.082×BMFB)	0.39	-0.41	0.710	0.025
mDF #4: Ramus only	−12.700 + (0.185×RH) + (0.140×SB) + (− 0.023×BMFB)	0.59	-0.63	0.821	0.021
mDF #5: Body fragment	−8.253 + (0.251×SH)	0.27	-0.29	0.798	0.022
mDF #6: Three most Dimorphic	−18.615 + (0.111×BGD) + (0.101×RH) + (0.018×MML)	0.75	-0.79	0.864	0.018
**Stepwise Multiple Variables**					
sDF #1	−37.506 + (0.183×MA) + (0.084×BGB) + (0.275×RH) + (0.189×MBL) + (− 0.196×MML)	0.87	-0.92	0.893	0.016

# Key to measurement in Table 2

#### Single variable demarking points against corresponding posterior probabilities

Demarking points corresponding to mid-points and posterior probability of 0.8, 0.9 and 0.95, relative to sex are summarized in Table 7 and visualized in Fig. 2 using a spider web plot. Increasing distance from the midpoint is associated with a larger posterior probability value.

**Table 7:** Single variable demarking points (in mm) for females and males corresponding to posterior probabilities of 0.95, 0.90, 0.80 and 0.50

Measurements ^#^	Female				Male		
	0.95	0.90	0.80	0.50	0.80	0.90	0.95
**BCoB**	76	80	85	97	100	110	117
**BGB**	81	85	88	96	104	108	111
**RH**	41	46	50	57	65	68	72
**MML**	100	105	114	123	132	135	140

# Key to measurement in Table 2

**Fig. 2:**
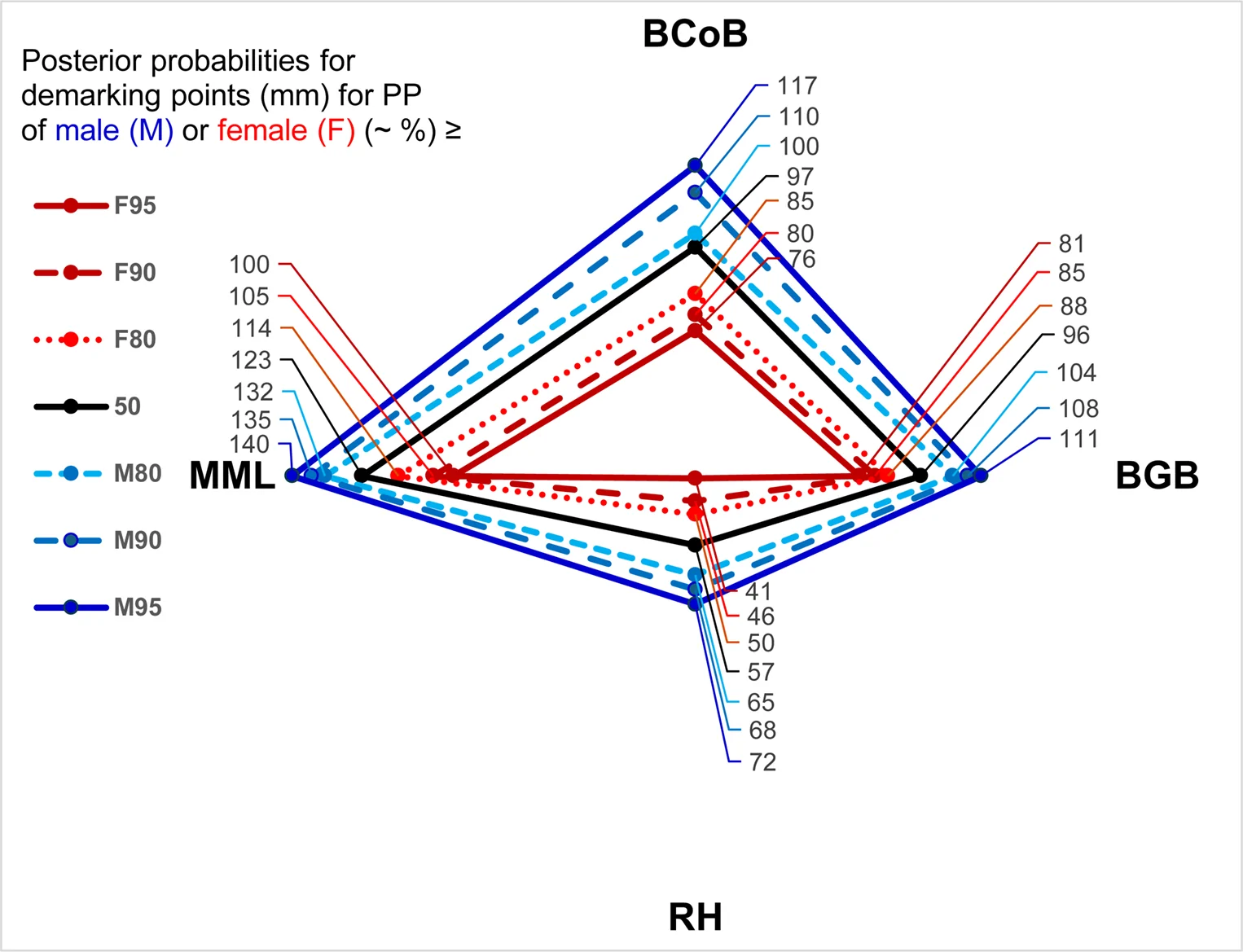
Spider web plot of single demarking points (in mm) of male (blue) and female (pink) measurement values against corresponding posterior probabilities of 50% (black line indicates mid-point between the two groups), 80%, 90% and 95%. RH: Ramus height; MML: Maximum mandible length; BCoB: Bicoronoid breadth; BGB: Bigonial breadth

#### Practical application of developed sex estimation models

To enhance the practical utility of the developed discriminant function models, their application are determined by the preservation status of the recovered mandible and the availability of measurable anatomical landmarks. Table 8 summarizes the recommended models, corresponding classification metrics, and suggested forensic applications.

**Table 8:** Practical Recommendations for Application of Mandibular Sex Estimation Models in the Contemporary Malaysian Population

Mandibular preservation	Model	Measurements Involved	Equations	Cross-Validated Accuracy (%)	Sex Bias (%)	Recommended Forensic Application
Complete mandible	sDF #1	MA, BGB, RH, MBL, MML	−37.506 + (0.183×MA) + (0.084×BGB) + (0.275×RH) + (0.189×MBL) + (− 0.196×MML)	83.60	-2.02	Recommended as the primary model when complete mandible is preserved. Demonstrates the highest classification accuracy among the models, strong classification confidence, and practical quantity of measurement involved, which balanced between practicality and accuracy. Interpret with other skeletal and contextual evidence.
Half Mandible	mDF #2	RH, CH, MA, SH, MML	−23.634 + (0.135×RH) + (0.035×CH) + (0.061×MA) + (0.008×SH) + (0.051×MML)	77.60	0.71	Should only be applied as complementary methods to other sex estimation methods.
Ramus only	mDF #4 (Ramus Only)	RH, SB, BMFB	−12.700 + (0.185×RH) + (0.140×SB) + (− 0.023×BMFB)	74.00	-3.44	Not recommended
Body Only	mDF #3	SH, SB, BMFB	−15.231 + (0.164×SH) + (0.252×SB) + (0.082×BMFB)	67.20	-5.01	Not recommended
Body Fragment	mDF #5	SH	−8.253 + (0.251×SH)	62.70	-0.43	Not recommended
Single-variable model	dDF #2	BGD	−16.334 + (0.170×BGD)	75.90	-2.58	Best-performing single-variable model; suitable for rapid preliminary assessment. Should only be applied as complementary methods to other sex estimation methods.

When a complete mandible is available, the stepwise discriminant function model (sDF #1), incorporating mandibular angle (MA), bigonial breadth (BGB), ramus height (RH), mandibular body length (MBL), and maximum mandibular length (MML), is recommended as the preferred model. This model achieved the highest cross-validated classification accuracy (83.6%) and demonstrated low sex bias (− 2.02%), while requiring a manageable number of measurements. Consequently, it provides an effective balance between classification performance and practical applicability.

In cases involving incomplete mandibular remains, model selection should be based on the preserved anatomical region. For specimens preserving approximately half of the mandible, the half-mandible model (mDF #2) is recommended, yielding a cross-validated accuracy of 77.6% and a sex bias of 0.71%. This model should be applied as supplementary method when complete mandibular measurements cannot be obtained.

For highly fragmented remains, classification performance decreases substantially. The ramus-region model (mDF #4) achieved a cross-validated accuracy of 74.0%, whereas the body-only (mDF #3) and body-fragment (mDF #5) models yielded accuracies of 67.2% and 62.7%, respectively. Accordingly, these models are not recommended for forensic investigation.

Among the direct single-variable models, bigonial breadth (BGD) demonstrated the highest classification performance, with a cross-validated accuracy of 75.9% and a sex bias of − 2.58%. This model may serve as a rapid preliminary screening tool. Nevertheless, single-variable approaches should not be considered substitutes for multivariate models and are best applied as complementary methods within a broader forensic assessment.

Based on the present findings, practitioners are encouraged to prioritize the use of multivariate models whenever sufficient anatomical preservation permits. Furthermore, mandibular sex estimation should be regarded as a supplementary component of biological profile construction and interpreted alongside other skeletal indicators, contextual information, and forensic evidence. 

## Discussion

The present study examined a documented sample of Malaysian cranial CT scans to establish a population-specific morphometric standard for sex estimation using the mandible. The mandible was selected for two reasons: firstly, this bone is frequently recovered largely intact (or sufficiently complete to provide diagnostic fragments) in contrast to other less dense skeletal elements [32]; and secondly, there is a notably limited availability of metric standards for the mandible specific to the Malaysian population.

Assessment of intra-observer error indicated rTEM values of less than 5% and R values larger than 0.75 across all measurements, which is compatible with the acceptable precision limit outlined by recognised anthropological research [23, 29]. Previous studies have shown that measurements acquired from volume rendered MSCT scans are equivalent to those obtained from physical bone specimens [23], therefore the models presented in this study are suitable for Malaysian forensic practice.

The present study demonstrated that ten of the 11 mandibular measurements analysed were significantly dimorphic; with bicoronoid breadth, bigonion breadth, ramus height and maximum mandible length being the most dimorphic. The latter mandibular measurements have been widely recognised among the most dimorphic in a range of different populations, such as Thai [33], Indonesian [34], Israeli [35], and Greek [31].

The mandibular angle has been shown to exhibit sexual dimorphism, with females often having a more obtuse (more than 120 degrees) and wider mandibular angle than males, who have a more rounded and acute angle [36]. In contrast, the means of the mandibular angle in this study did not differ significantly between sexes; a finding similar to that in a South Indian population [9]. Similar results were observed in a young adult of Lebanese population, which showed that there are no differences in mandibular angle from lateral cephalometric radiographs [37]. Within Southeast Asian population, our data accords with the findings from an Indonesian population, which also showed no significant difference between sexes for the mandibular angle [38].

The mandibular measurements demonstrated consistently larger dimensions in males compared to females in present study. The differences in sexual dimorphism observed in the mandible are primarily attributed to hormonal, functional and biomechanical factors that influence craniofacial growth and development [39, 40]. Functionally, mandibular dimorphism is influenced by the development and mechanical loading of the masticatory muscles, such as masseter, temporalis, medial and lateral pterygoids, which are inserted to the ramus, condyle, symphysis and coronoid process [41]. For example, males generally exert greater bite force and exhibit more hypertrophied muscles of mastication, especially the masseter and temporalis, which induce stronger biomechanical stresses on the mandibular body and ramus [41]. Over time, these mechanical demands stimulate bone remodelling in accordance with Wolff’s Law, leading to a bony structural changes [42]. Therefore, condyle and ramus exhibit the most sexually dimorphic morphological changes [43].

Population-specific standards in forensic anthropology are essential for enhancing the accuracy of demographic estimations [15, 44]. Applying anthropological standards established for geographically distinct populations increases the probability of misclassification [45]. Furthermore, the sex bias also potentially increases, indicating differences in the expression of sexual dimorphism within and between populations. This study demonstrated that when applying formulas developed for foreign, non-Malaysian groups to a Malaysian population, the accuracy of sex estimation is notably reduced. Application of Malaysian mandibular measurements into model from Chinese Han population had the accuracy dropped massively to 61.84% with sex bias increased to -66.44%, much more significantly compared to the South African or Greek models. Despite geographic and ancestral proximity between Chinese Malaysian and Chinese Han population, it does not guarantee similarities in skeletal morphology. The Malaysian population examined in this study comprises a multi-ethnic cohort, including Malay, Chinese, and Indian individuals, with the Chinese subgroup constituting approximately 30% of the sample, reflecting a more heterogeneous population compared to the predominantly Han Chinese reference sample, reflecting a more homogeneous population used by Dong et al. This difference in population composition may contribute to the reduced transferability of the model, as discriminant functions derived from relatively homogeneous populations may not perform optimally when applied to more diverse cohorts. In addition, the disparities in skeletal measurements across different populations can be attributed to other factors such as climate, diet, genetic and physical activity [46].

Four univariate discriminant functions were generated for the Malaysian population, with accuracy ranging from 70.40 to 75.90%. Bigonial breadth demonstrated the highest accuracy with an associated sex bias of -2.58%. These findings align with those from non-Malaysian populations, where bigonial breadth has commonly been recognised as a highly dimorphic feature of the mandible. Classification accuracy rates of bigonial breadth for sex estimation were reported as 70.7% for South African [6], 73.2% for Greek [31], and 70% for Thai population [33]. None of the univariate discriminant functions have achieved a cross-validated accuracy of greater than 80% and a sex bias of less than 5%, implying that reliance on a single metric variable may be insufficient for accurate and reliable estimation of sex in Malaysian population. Consequently, these univariate measurements should be interpreted with caution when used independently for forensic sex estimation, which reinforces the necessity of the multivariate models [47].

Although model mDF #6 demonstrated a relatively high overall classification accuracy (80.7%) among multivariate models, only approximately 53% of the sample achieved a posterior probability (PP) greater than 0.80. This indicates that nearly half of the classifications fall within a lower confidence range, which has important forensic implications. In a practical forensic context, classifications with low posterior probabilities are generally considered insufficiently reliable and may be treated as indeterminate rather than definitive. This highlights that overall accuracy alone may overestimate the practical utility of a model if a substantial proportion of cases are associated with low confidence. Similarly, while sDF #1 achieved the highest classification accuracy and posterior probability, mDF #1 demonstrated slightly superior performance in terms of area under the curve (AUC = 0.90), indicating stronger descrimination across thresholds. While both models demonstrated classification accuracy exceeding 80%, sDF #1 practically advantageous as it requires only five variables, thereby enhancing efficiency and ease of application. Notably, sDF #1 maintains a favourable balance between overall accuracy, posterior probability distribution, and classification performance, despite exhibiting a slightly lower area under the curve (AUC) compared to mDF #1. This distinction underscores the importance of reporting AUC and posterior probabilities with classification accuracy to facilitate informed interpretation relative to forensic practice.

The results of the stepwise discriminant analysis combining mandibular angle, bigonial breadth, ramus height, maximum body length and maximum mandibular length achieved overall the highest accuracy (83.6%) with a sex bias of -2.02%. The accuracy derived from this analysis is comparable to the discriminant functions from other population samples, such as 81.1% in an Indian population [48] and 81.8% in a South African population [6]. The only two other discriminant functions achieving a cross-validated classification accuracy greater than 80% are the functions derived from the complete mandible using all measurements (82.4%, sex bias − 0.51%), and the combination of the three most dimorphic features (80.7%, sex bias 0.06%). These accuracy values are slightly below the those reported from other populations, such as the South African sample, which demonstrated an accuracy of 84% for complete, and 82.7% for half, mandibles [6]. In comparison, an Israeli sample reported an accuracy of 90.8% for the complete mandible and 85.6% for the half mandible [35]. The accuracy value for the half mandible in the present sample was 77.6% (with a sex bias of 0.71%). The combination of measurements for fragmentary mandibles presented in this study have not achieved the accepted thresholds for forensic application, and therefore should only be applied as complementary methods to other sex estimation methods. The spiderweb plot presents a novel approach to provide Malaysian forensic practitioners with a simple visual tool for a quick preliminary estimation of sex using the most dimorphic single measurements and including posterior probabilities of being male or female.

When compared with other postcranial skeletal elements studied within the Malaysian population, the mandible demonstrates a classification performance within the moderate to high accuracy range. Previous Malaysian studies have reported sex estimation accuracies of 80.3% for the scapula using three-dimensional geometric morphometric methods, and 87.9% for the atlas using two-dimensional geometric morphometrics [49]. In addition, combined long bone models incorporating the femur and humerus have achieved higher accuracies values of up to 91.8% using discriminant function analysis [50]. Smaller postcranial elements such as phalanges have also demonstrated moderate performance, with reported accuracies of approximately 81.8% [51]. In comparison, the mandibular models developed in the present study achieved an overall accuracy of 83.6%, placing them within a similar performance range as postcranial elements. These findings suggest that, within the Malaysian population, the mandible provides a comparable level of classification performance to several established skeletal indicators, despite generally being considered a secondary sexually dimorphic structure. Importantly, the present study extends beyond previous work by additionally evaluating how classification performance varies according to different mandibular fragments and measurement subsets, thereby providing a more comprehensive understanding of mandibular utility in forensic contexts where complete mandibles may not be available. Furthermore, the introduction of spider web plots illustrating single demarking points across multiple posterior probability thresholds enhances interpretability and offers a practical visual tool for forensic casework, which is rarely addressed in existing studies.

Despite the promising outcomes of this study, several limitations warrant consideration.

First, the sample size, although representative of the contemporary Malaysian population, may not fully capture the country’s diverse composition. Malaysia’s population comprises Malay, Chinese, Indian and various Indigenous groups, with each potentially exhibiting a different magnitude and expression of sexual dimorphism in mandibular morphology. Consequently, while the present models demonstrate robust discriminatory accuracy, their applicability across all subpopulations should be interpreted with caution. Second, the study used a combined sample of adult individuals, while age-related morphological changes in the mandible were not considered. The effect of age on the mandible (such as tooth loss and resorption of the soft and hard tissues of the mandible) may influence the accuracy of sex estimation in elderly and needs to be explored. Thirdly, the applicability of population-specific sex estimation models may be limited in forensic scenarios where the population affinity of unknown remains cannot be confidently established, such as mass fatality incidents involving individuals from diverse backgrounds. Consequently, the discriminant functions developed in this study should not be viewed as universally applicable standards. Rather, they are intended to provide population-relevant reference data for the contemporary Malaysian population and to contribute to the growing body of evidence regarding population variation in mandibular sexual dimorphism. Furthermore, sex estimation should ideally be based on the integration of multiple skeletal elements and complementary lines of forensic evidence. The mandibular models presented herein are therefore best regarded as supplementary tools that may assist biological profile reconstruction when more sexually diagnostic skeletal regions, such as the pelvis, are unavailable, fragmented, or incomplete. Future research should expand the dataset to encompass a broader representation of Malaysia’s population diversity and investigate on inter-ethnic differences. Combined cranial and mandibular (skull) models may be explored to further enhance classification performance. Age-stratified analyses are also recommended to elucidate changes to mandibular dimorphism due to age. Furthermore, in line with recent advancements in forensic anthropology, the application of machine-learning, deep-learning approaches or web-based huMANid tools application should be considered, either as alternatives or in combination with traditional discriminant function analysis. Such approaches may improve predictive performance by capturing complex, non-linear relationships within the data, and could provide valuable complementary insights to conventional statistical models.

## Conclusion

This study formulated and validated discriminant functions for sex estimation using mandibular measurements of a contemporary Malaysian population. The resultant population-specific models were more accurate and with lower sex bias values compared to previously published discriminant functions based on non-Malaysian populations. The latter represents an important to the literature because it further reinforces the significance of population-specific approaches in forensic practice. Among the Malaysian-specific discriminant functions, only analysis of the complete mandible achieved sufficient discriminatory accuracy to be considered applicable for forensic investigation. The discriminant functions derived from partial or fragmentary mandibular measurements, while informative, exhibited lower classification accuracy and may therefore have limited standalone forensic utility. However, the integration of mandibular data with complementary cranial or pelvis data will enhance the accuracy of biological profiling. This study provides a crucial foundation for understanding mandibular sexual dimorphism within the heterogeneous Malaysian population and underscores the need for continued research exploring intra- and inter-population variation and their implications for forensic application.
